# Salinity-Based Toxicity of CuO Nanoparticles, CuO-Bulk and Cu Ion to *Vibrio anguillarum*

**DOI:** 10.3389/fmicb.2017.02076

**Published:** 2017-10-25

**Authors:** Alice Rotini, Andrea Tornambè, Riccardo Cossi, Franco Iamunno, Giovanna Benvenuto, Maria T. Berducci, Chiara Maggi, Maria C. Thaller, Anna M. Cicero, Loredana Manfra, Luciana Migliore

**Affiliations:** ^1^Department of Biology, University of Rome Tor Vergata, Rome, Italy; ^2^Institute for Environmental Protection and Research (ISPRA) Rome, Italy; ^3^Qi Technologies Pomezia, Rome, Italy; ^4^Research Infrastructures for Marine Biological Resources, Stazione Zoologica Anton Dohrn, Naples, Italy; ^5^Department of Biology and Evolution of Marine Organisms, Stazione Zoologica Anton Dohrn, Naples, Italy

**Keywords:** metal oxide, marine bacteria, bioassay, nanoparticle behavior, copper dissolution, salinity influence

## Abstract

Bacteria are used in ecotoxicology for their important role in marine ecosystems and their quick, reproducible responses. Here we applied a recently proposed method to assess the ecotoxicity of nanomaterials on the ubiquitous marine bacterium *Vibrio anguillarum*, as representative of brackish and marine ecosystems. The test allows the determination of 6-h EC_50_ in a wide range of salinity, by assessing the reduction of bacteria actively replicating and forming colonies. The toxicity of copper oxide nanoparticles (CuO NPs) at different salinities (5-20-35 ‰) was evaluated. CuSO_4_ 5H_2_O and CuO bulk were used as reference toxicants (solubility and size control, respectively). Aggregation and stability of CuO NP in final testing dispersions were characterized; Cu^2+^ dissolution and the physical interactions between *Vibrio* and CuO NPs were also investigated. All the chemical forms of copper showed a clear dose-response relationship, although their toxicity was different. The order of decreasing toxicity was: CuSO_4_ 5H_2_O > CuO NP > CuO bulk. As expected, the size of CuO NP aggregates increased with salinity and, concurrently, their toxicity decreased. Results confirmed the intrinsic toxicity of CuO NPs, showing modest Cu^2+^ dissolution and no evidence of CuO NP internalization or induction of bacterial morphological alterations. This study showed the *V. anguillarum* bioassay as an effective tool for the risk assessment of nanomaterials in marine and brackish environments.

## Introduction

The metal nanoparticles, including metal oxides, represent one of the major classes of commercial nanomaterials, which are manufactured on a large scale for both industrial and household applications (Chang et al., 2012). Copper (II) oxide nanoparticles (CuO NPs) are increasingly used in several products (Huang et al., 2010; Chang et al., 2012; Rossetto et al., 2014). The wide variety of applications entails the risk of environmental contamination, as a consequence of the environmental release of CuO NP during their production, use and disposal (Weinberg et al., 2011; Sanchís et al., 2013; Fan et al., 2014). This kind of contamination could bias both organisms and ecosystems (Gambardella et al., 2013), as CuO NPs can exert toxic effects but also antimicrobial activity on (environmental) microbes (Bondarenko et al., 2013; Rossetto et al., 2014); hence, they could seriously affect estuarine and coastal environments, considered the ultimate sink for different kinds of NPs (Canesi et al., 2012). Accordingly, the investigation on CuO NP effects in the brackish/marine ecosystems has become a very important issue, and must include information on NP fate, transport and toxicity (Lowry et al., 2012).

Nevertheless, ecotoxicity of CuO NPs, particularly to marine organisms, is still little explored and the available data are often inconsistent, due to different and no standardized experimental approaches that highlighted some procedural limitations, mainly related to the stability of nanomaterials during the test exposure (reviewed by Minetto et al., 2016). The great majority of the studies evaluated endpoints as oxidative stress, genotoxicity, bioaccumulation and behavioral impairments (Bondarenko et al., 2013; Ivask et al., 2013; Minetto et al., 2016; Gonçalves et al., 2017) or soil toxicity (Unrine et al., 2010; Amorim and Scott-Fordsmand, 2012; Amorim et al., 2012; Gomes et al., 2012; Gomes S. I. et al., 2015; Gomes S. I. L. et al., 2015; Gonçalves et al., 2017), with few EC_50_ identifications, although it is mandatory to define the hazard of CuO NPs in environment and human health and enhance their safe use.

Bacteria are an important component of brackish/marine ecosystems and alterations of the microbial communities could have significant effects on biogeochemical cycling and other critical ecosystem services. Toxicity tests based on microorganisms are gaining popularity even because they are relatively quick, reproducible, cheap and do not imply ethical issues (Parvez et al., 2006). Among bacterial bioassays, *V. fischeri* luminescence inhibition test, based on the enzymatic activity of the bacterial luciferase, is the most common and well-standardized one (Azur Environmental, 1995). However, this bioassay has some constrains even for conventional contaminants: for example, it cannot be utilized with samples at salinities exceeding a quite narrow range (APAT IRSA-CNR, 2003), or with colored and turbid samples, due to possible interferences with luminescence measurements. As regard other bioassays with bacterial species (*B. subtilis, E. coli, L. brevis* and *S. aureus*), most of them are based on the same endpoint, i.e., the inhibition of replication rate of the bacterial culture, but they use different and no standardized protocols (Baek and An, 2011; Kaweeteerawat et al., 2015; Bondarenko et al., 2016). These methods present some criticisms that can influence the NP toxicity assessment and affect the repeatability of the bioassay. For instance, the possible interactions between NPs and organic matter in the exposure medium, the excessively reduced test volumes, without any mixing during the exposure, or the need of correction factors for colored/turbid samples.

In order to provide a useful tool for NP toxicity assessments in marine environments, we recently developed a new bioassay with the marine bacterium *Vibrio anguillarum* and demonstrated its effectiveness in evaluating the toxicity of a reference toxicant (Rotini et al., 2017). The model organism *V. anguillarum* is a Gram-negative, short curve-shaped rod bacterium with a polar flagellum. It was chosen because of its intrinsic characteristics: it is halotolerant, ubiquitous and plays important ecological roles in marine/brackish ecosystems (Thompson et al., 2004). The bioassay allows to assess the decrease of bacterial culturability and to determine the EC_50_ (i.e., the concentration causing the 50% reduction of bacteria actively replicating and forming colonies, after 6-h exposure).

In this study, the suitability of this bioassay in evaluating NP toxicity has been checked. To this end, the study evaluated and compared the ecotoxicity of CuO NPs, CuO-bulk and Cu^2+^ ion (CuSO_4_ 5H_2_O) in a wide range of salinity (5–35‰), by using the recently proposed test on the marine bacterium *V. anguillarum*. To deepen the CuO NP behavior during the test and relate it to toxicity, the physicochemical characterization of NPs in the exposure medium was carried out and the size distribution, sedimentation rates and Cu^2+^ dissolution from NPs were analyzed. These data allow a better understanding of NP aggregation dynamics and stability at the different salinities. Furthermore, to provide the most accurate picture, even CuO NP internalization or morphological alterations were evaluated in bacteria at the end of the test exposure.

## Materials and methods

### Reagents and solutions

0.5-2-3.5% NaCl solutions were prepared as exposure media by dissolving NaCl (Sigma-Aldrich, pure grade) in deionized water. Tryptic Soy Agar (TSA, Liofilchem, 40 g/L) and Tryptic Soy Broth (TSB, Liofilchem, 30 g/L) growth media for bacteria were prepared in deionized water adding the appropriate amount of NaCl to obtain the same salinity of the exposure medium. NaCl solutions, TSA and TSB media were sterilized (121°C, 15′). CuO NPs (25–55 nm size) were purchased by US Research Nanomaterials, Inc. shipped as ultrapure water dispersion (20% w/v, purity of 99.95%). CuO NP stock dispersion (1 g/l) was prepared in 0.22 μm filtered milli-Q water (mQW) from the 20% dispersion after 15 min of sonication (1210E-MT Branson ultrasonic bath) at 60 w and 47 kHz. CuO NP stock dispersion was sonicated for 15 min, stored in the dark at 4 °C and used for preparation of all the final testing dispersions. CuO NP final dispersions were prepared from the stock dispersion, previously sonicated for 15 min. CuSO_4_ 5H_2_O (Sigma-Aldrich, purity ≥ 98%) was used as positive and solubility control; CuSO_4_ 5H_2_O stock solution (400 mg/l) was prepared in deionized water and the necessary aliquots were sterilized by using a 0.22 μm syringe filter. CuO-bulk (micrometric particles of CuO) was purchased by US Research Nanomaterials Inc. to be used as size control (Schultz et al., 2014); CuO-bulk stock (8 g/l) and final dispersions were prepared following the same procedure described above for CuO NPs.

For ICP-MS measurements, stock Cu standard solution (1,000 μg/l) and HNO_3_ Ultrapur were purchased from Romil. Calibration standards were prepared within a linear range (2.5–40 μg/l) from the stock Cu standard solution in 0.5 M HNO_3_. Standard solutions were freshly prepared and standard calibration curves with *R*^2^ = 0.99998 were achieved daily.

### CuO NP characterization

The CuO NP stock dispersion (1 g/l), in milli-Q water, was analyzed via SEM to characterize particle sizes and shapes. The stock dispersion was diluted 1/100, placed on a membrane filter of 0.2 μm-pore size and platinum sputter coated (Polaron SC7640, Quo-rum Technologies Ltd., Ashford, UK). Stubs were observed with a Field Emission Scanning Electron Microscope JSM6700F (JEOL, Ltd, Tokyo, Japan).

Two different characterization techniques were used to estimate the particle size distributions and stability in the final testing dispersions at different salinities: Analytical Centrifugation (LUMISizer, L.U.M. GmbH, Berlin) and DLS (NICOMP 380 DLS Particle Size Analyzer, PSS, FL USA). The size distribution of the CuO NPs in the saline solutions (0.5-2.0-3.5% NaCl) and in the reference medium (mQW), was measured by DLS, with a 658 nm wavelength 30 Mw laser and a 90° scattered light Avalanche Photo Detector. Two readings of 5 min per sample were acquired and data processed following the NICOMP algorithm that automatically selects the best fitting distribution and recognizes from one to three particle size populations. The dimensional analyses for each population calculated the volume-weighted diameters (±standard deviation) and the relative percentages. Zeta (ζ-) potential of the CuO NPs in the saline solutions (0.5-2.0-3.5% NaCl) and in the reference medium (mQW) was also measured by DLS. Measurements were carried out in triplicate, each consisting in 7 runs. The average particle sedimentation velocity as well as the particle size distribution (PSD) were also investigated (according to ISO 13318-2, 2007) by the Dispersion Analyser LUMiSizer. This instrument consists of an analytical centrifuge with an optoelectronic sensor system. It measures variations in the transmitted near infra-red radiations along horizontally inserted sample tubes, due to the sedimentation of suspended material. The integration of transmission profiles, sedimentation rates and particle size distributions were calculated by using the LUMiSizer software, SEPView 6.3.

The amount of metal dissolution from the CuO NPs into the exposure medium has been investigated. A CuO NP final dispersion (40 mg/l), at different salinities (0.5-1.5-2.0-3.5% NaCl), was centrifuged at 4,000 × *g* (centrifuge PK121R, ALC International S.r.l., Italy) for 60 min to remove the non-soluble fraction of CuO. The concentration of Cu ions was quantified by inductively-coupled plasma mass spectrometry (ICP-MS 7900 Agilent) according to USEPA 6020b (2014). Control and centrifuged samples were analyzed in triplicate after acidification with HNO_3_ s.p. (0.5% v/v).

### Ecotoxicity test

*Vibrio anguillarum* (strain AL 102, serotype O1; from NOFIMA collection) was exposed to *five* concentrations of three Cu forms (CuO NP, CuO bulk and CuSO_4_ 5H_2_O) in saline solution (no growth medium), to evaluate the reduction of the bacterial culturability (i.e., the capability to replicate and form colonies) after 6-h exposure, according to the protocol shown in Rotini et al. (2017). A liquid fresh culture of *V. anguillarum* was used to obtain the bacterial inoculum. After the overnight incubation (12–18 h, 25 °C) the bacterial concentration of the inoculum was estimated spectrophotometrically (UV/Visible Spectrophotometer Beckmann 473) at 600 nm and diluted to an OD value of 0.14 (corresponding to the 0.5 point of McFarland nephelometric standard). The diluted inoculum was then centrifuged for 10 min at 3,000 *g*. The bacterial pellet was resuspended in 1 ml of exposure medium (saline solution) and 150 μl were added to each test tube, including the control, in a final volume of 5 ml. Control and test dispersions were incubated for 6 h at 25°C, in the dark with continuous shaking (120 rpm), to avoid sedimentation. At the beginning (T_0_) and the end (T_6_) of the exposure time, bacterial counts in all the CuO NP dispersions and control were evaluated, by using the liquid-to-plate micro-counting method (Migliore et al., 2013; Rotini et al., 2017). Briefly, it consists in preparing serial dilutions of each exposed bacterial suspension, applying a ten-fold dilution factor (up to 10^87225^). A small aliquot (10 μl) of dilutions is plated on TSA Petri dishes, then incubated at 25°C for 48 h. Colonies grown on petri dishes were counted and results were used to estimate the number of Colonies Forming Units per ml (CFU/ml). The counting from three replicate plates for each toxicant concentration and control were used to evaluate the mean number of bacteria actively replicating and forming colonies. Three independent tests, (using a freshly prepared bacterial inoculum for each test) at each of the three different salinities (0.5-2-3.5% NaCl) were performed on and five concentrations of each chemical (CuO NPs: 10-20-40-80-160, CuO bulk: 80-160-320-640-1280, CuSO_4_ 5H_2_O: 0.625-1.25-2.5-5-10, mg/l), i.e., 9 tests each time, as a total of 27 tests. The concentrations were chosen as a result of preliminary tests. In these tests the toxicants showed very different toxicity, hence, for each toxicant, the final testing concentrations were chosen to allow the calculation of the Effect Concentration (EC_50_).

The EC_50_, i.e., the Effect Concentration of toxicant that reduces of 50% the number of bacteria actively replicating and forming colonies, after 6-h exposure, was calculated by non-linear regression (Log-Normal model) of the mean number of CFU/ml for each concentration, by using R software, drc package (Ritz and Streibig, 2005). Significant differences among treatments were evaluated by using one-way analysis of variance (ANOVA) followed by *post-hoc* pairwise *t*-tests (R software, stats package).

### Scanning electron microscope (SEM) analysis

After exposure, bacteria were centrifuged at 3,000 *g* for 10 min and fixed in 2% glutaraldehyde in 2% NaCl solution. After 24 h, fixed bacteria were rinsed three times with PBS 1X and post-fixed with 1% osmium tetroxide, at 4°C for 1 h. After three washes with bi-distilled water, bacteria were placed on a membrane filter of 0.4 μm-pore size in a Swinnex filter holder (Millipore, Billerica, Massachusetts, USA). Samples were then washed with 10 mL of bi-distilled water for approximately 30 min and dehydrated in a graded ethanol series. The sample was critical point dried, platinum sputter coated (Polaron SC7640, Quo-rum Technologies Ltd., Ashford, UK) and observed by a field emission SEM, JEOL JSM 6700F (JEOL Ltd, Tokyo, Japan).

### Transmission electron microscope (TEM) analysis

After exposure, bacteria were centrifuged at 3,000 *g* for 10 min and fixed in 2% glutaraldehyde in 2% NaCl. After 24 h, fixed bacteria were rinsed three times with PBS1X and post-fixed with 1% osmium tetroxide, at 4°C for 1 h, rinsed five times with bi-distilled water, dehydrated in a graded ethanol series, further substituted by propylene oxide, embedded in Epon 812 (TAAB, TAAB Laboratories Equipment Ltd, Berkshire, UK) and kept at room temperature for 1 day and then polymerized at 60°C for 2 days. Resin blocks were sectioned with an Ultracut UCT ultramicrotome (Leica, Vienna, Austria). Ultrathin sections (50–60 nm) were placed on nickel grids, contrasted with 4% aqueous uranyl acetate for 30 min, rinsed once with a mix of methanol and bi-distilled water (1:1), twice with bi-distilled water and observed by a TEM Zeiss LEO 912AB (Zeiss, Oberkochen, Germany).

## Results

### CuO NP characterization

**Figure 1:**
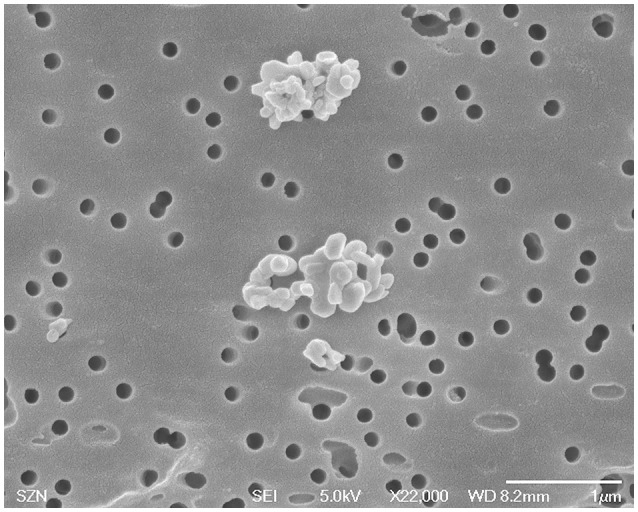
Characterization of CuO NPs used in this study in reference medium (milli-Q water) by SEM analysis.

The CuO NP stock dispersion, in milli-Q water, has been characterized by SEM (Figure 1). Nanoscale spherical particles (about 50 nm) were present, confirming particle sizes provided by the manufacturer; microscale aggregates with diameters ranging from about 100 to 500 nm were also observed.

The Zeta (ζ-) potential and average volume-weighted diameters (Table 1) of the CuO NP dispersions were measured by DLS in the reference medium (mQW) and the saline solutions (0.5-2.0-3.5% NaCl) used as exposure media (see Figure S1).

**Table 1:** Characterization of CuO NP dispersions in milli-Q water (mQW, T = 25°C) and three saline solutions used as exposure media (*T* = 25 °C, 0.5-2.0-3.5% NaCl) using DLS analysis.

	**Peak 1 - D (nm)**	**%**	**Peak 2 - D (nm)**	**%**	**ζ-potential (mV)**
mQW	78.7 ± 26.9	41.28	191.9 ± 44.2	58.72	−15.2 ± 2.0
0.5% NaCl	74.6 ± 16.0	68.57	247.8 ± 48.7	31.43	−2.5 ± 0.3
2.0% NaCl	211.7 ± 34.8	100	−	–	−1.4 ± 0.2
3.5% NaCl	313.1 ± 56.5	100	−	–	−1.7 ± 0.5

*Vol-wt diameters (D), relative percentage of each peak (%) and Zeta (ζ) potential are reported. Data are referred to 40 mg/l of CuO NP and values are average ± standard deviation of 3 measurements*.

**Figure 2:**
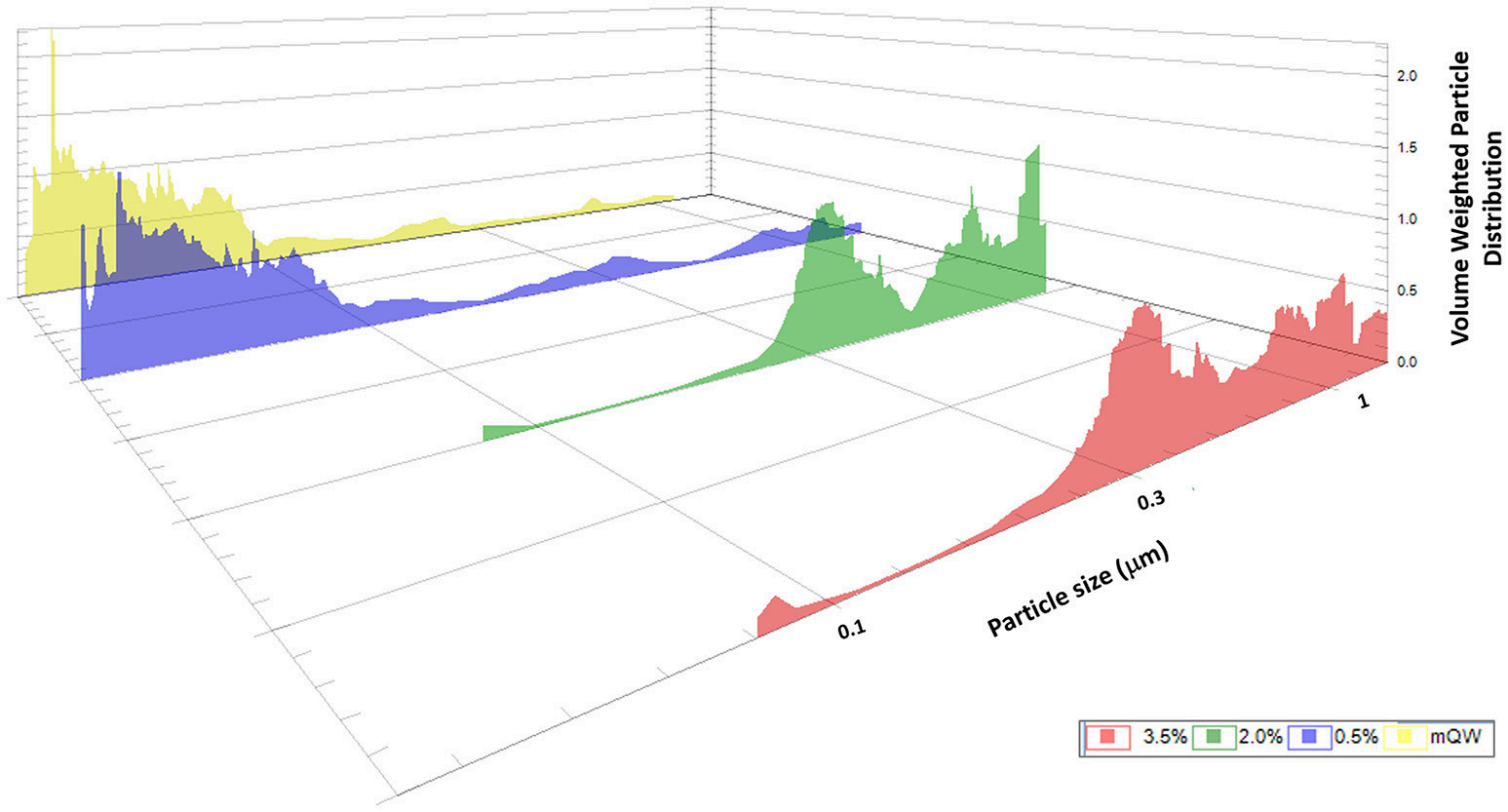
Characterization of CuO NP dispersions in milli-Q water (mQW, T = 25°C, salinity 0%) and three saline solutions used as exposure media (T = 25°C, 0.5%-2.0%-3.5% NaCl) using LUMISizer. Vol-wt particle size distributions referred to 40 mg/l of CuO NP are showed.

The average sedimentation rate of CuO NP agglomerates in the reference medium (mQW) was comparable to that measured in the 0.5% saline solution: 0.34 mm/h and 0.81 mm/h, respectively (see Figure S2). While, the average sedimentation rates of CuO NP agglomerates in 2.0 and 3.5% saline solutions were 2.52 and 2.85 mm/h, respectively. These high values account for the big sizes of agglomerates at the highest salinities. The particle size distribution in the exposure and reference media, returned by the LUMISizer analysis, is shown in Figure 2.

The Cu^2+^ concentration dissolved in the solution was limited, assuming a reduced release of ions from the CuO NP. The dissolved Cu^2+^ content slightly decreased with increasing salinity (see Table S1).

### Ecotoxicity tests

**Figure 3:**
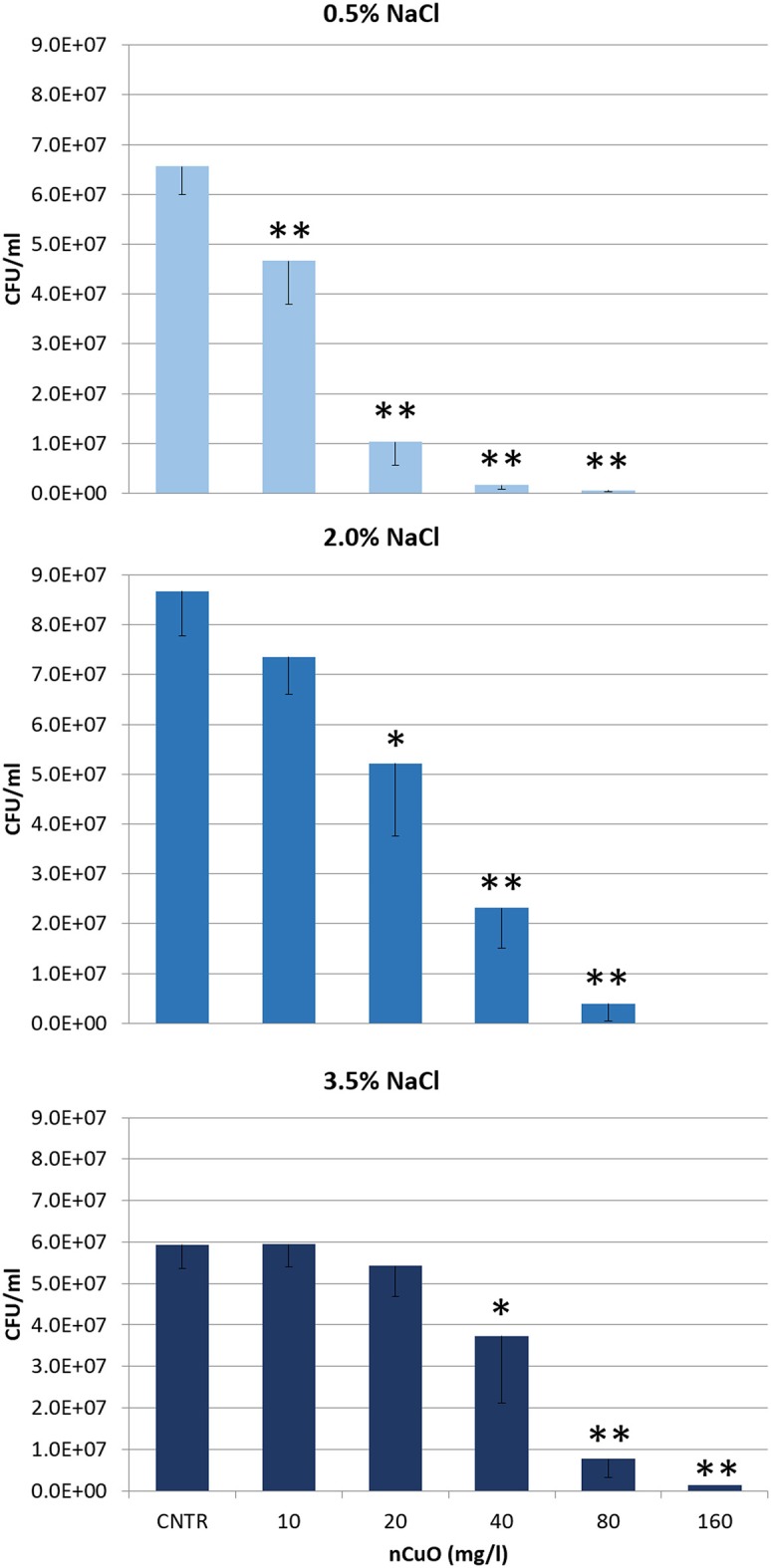
Mean number of CFU/ml (CFU = Colony Forming Unit) of *Vibrio anguillarum* after 6-h exposure to different concentrations of CuO NPs in exposure medium at three different salinities (*T* = 25°C, 0.5-2.0-3.5% NaCl). Values represent the mean of three independent trials; error bars represent standard deviation. Significant reduction of CFU/ml compared to control, based on *post hoc t*-test, are indicated with asterisks (*p < 0.05; **p < 0.01).

The results obtained from the exposure of *V. anguillarum* to the CuO NPs showed a clear dose-response relationship, although the toxicity changed according to both the concentration of CuO NPs and the salinity of the medium. In fact, all the tests showed inhibition of bacterial culturability, i.e., the capability to replicate and form colonies (measured as number of CFU/ml), at increasing NP concentration. However, a progressively reduced inhibition was found as salinity increases (Figure 3). Consequently, the average EC_50_ of CuO NPs increased with salinity (Table 2).

**Table 2:** Mean effect concentration (EC_50_) (mg/l) and 95% Confidence Limits of CuO NP, CuSO_4_ 5 H_2_O and CuO bulk calculated from three tests at three different salinities (*T* = 25°C, 0.5-2.0-3.5% NaCl).

	**%NaCl**	**Test 1**	**Test 2**	**Test 3**	**Geometric mean**
	0.5	11.7 (10.4–13.0)	12.4 (11.7–13.1)	13.7 (12.8–14.7)	12.6 (11.6–13.6)
CuO NP	2.0	24.3 (20.0–28.5)	26.3 (22.6–30.1)	20.5 (17.5–23.6)	23.6 (19.9–27.2)
	3.5	55.0 (41.6–68.3)	40.9 (36.9–44.8)	37.7 (29.8–45.6)	43.9 (35.7–51.9)
	0.5	1.2 (1.1–1.3)	1.2 (1.1–1.3)	1.3 (1.1–1.5)	1.2 (1.1–1.4)
CuSO_4_	2.0	1.0 (0.8–1.1)	1.0 (0.8–1.1)	0.8 (0.7–0.9)	0.9 (0.8–1.0)
	3.5	1.6 (1.4–1.8)	1.4 (1.2–1.7)	1.2 (1.0–1.4)	1.4 (1.3–1.8)
	0.5	241.0 (206.7–275.3)	223.1 (193.5–252.7)	231.8 (210.6–253.0)	231.9 (200.0–263.8)
CuO bulk	2.0	194.1 (177.4-210.8)	248.9 (238.0–259.8)	175.4 (130.9–219.9)	203.9 (176.8–229.2)
	3.5	182.0 (164.1-199.8)	222.1 (200.4–243.8)	184.1 (97.6–270.7)	195.2 (147.5–236.2)

The results obtained from the exposure of *V. anguillarum* to the solubility and size controls also showed a clear dose-response relationship. The solubility/positive control (CuSO_4_ 5H_2_O) showed a significant and progressive reduction of culturability, at increasing concentration of the toxicant (ANOVA, *p* < 0.001; see Figure S3). The number of CFU/ml is significantly reduced at 1.25 mg/l (*post hoc t*-test, *p* < 0.01), compared with control, regardless the medium salinity. Similarly, the size control (CuO bulk) showed a significant decrease of culturability with increasing concentration of the toxicant (ANOVA, *p* < 0.001) (see Figure S4). The number of CFU/ml is significantly reduced always at 320 mg/l (*post hoc t*-test, *p* < 0.01), compared with control and regardless the medium salinity. The average EC_50_ of solubility and size controls remained quite constant at different salinities (see Table 2).

### Microscopy analyses

**Figure 4:**
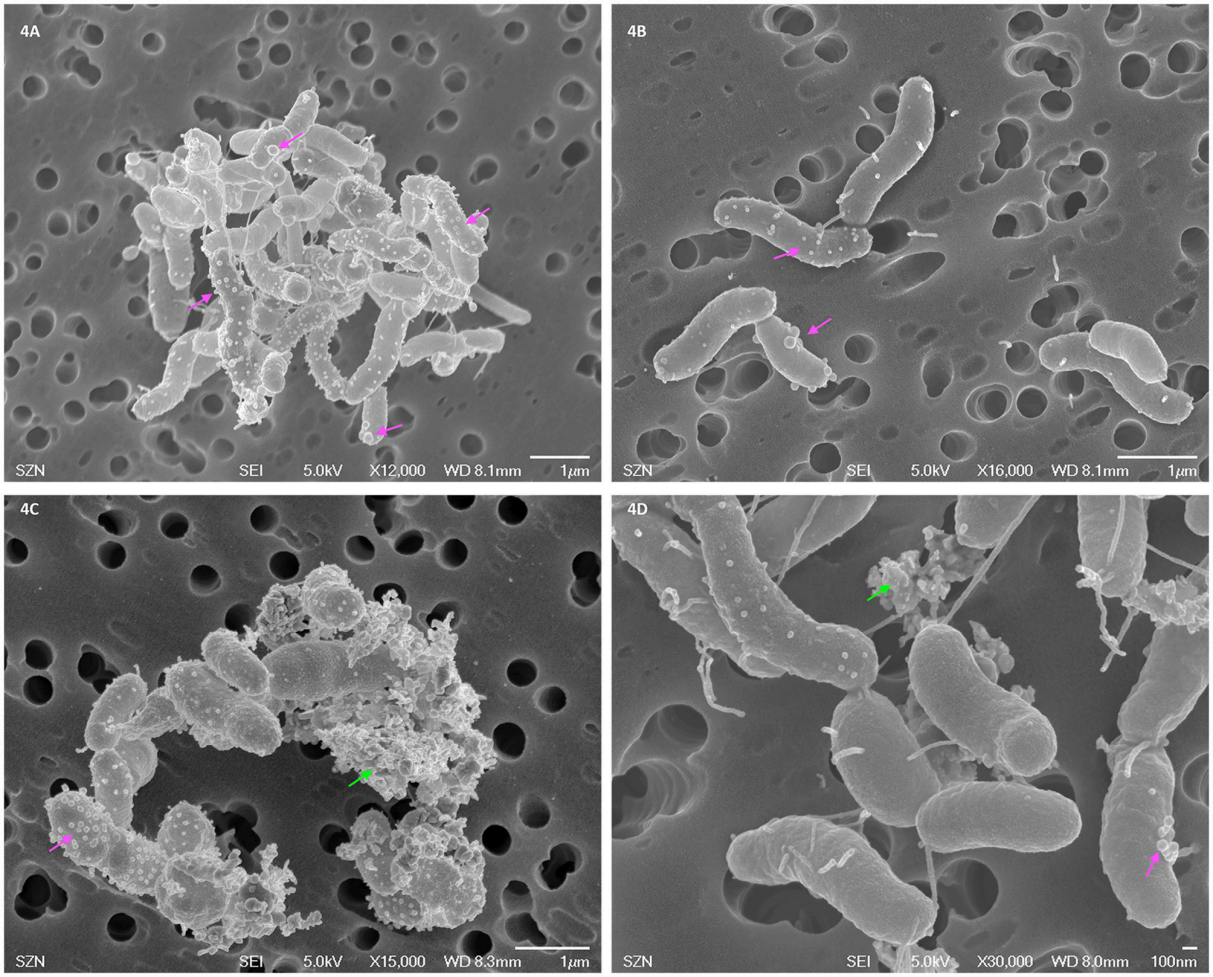
SEM images of *Vibrio anguillarum* after toxicity testing exposure in 2% NaCl saline solution: control **(A,B)** and CuO NP dispersion (40 mg/l, **C,D**). Blebs are indicated by pink arrows, nanoparticle aggregates by green arrows.

The Scanning Electron Microscope (SEM) analysis (Figure 4) did not highlight morphological differences between control and CuO NPs exposed bacteria. In both batches, blebs of different size and fibrils can be observed on the surface of the microbial cells.

**Figure 5:**
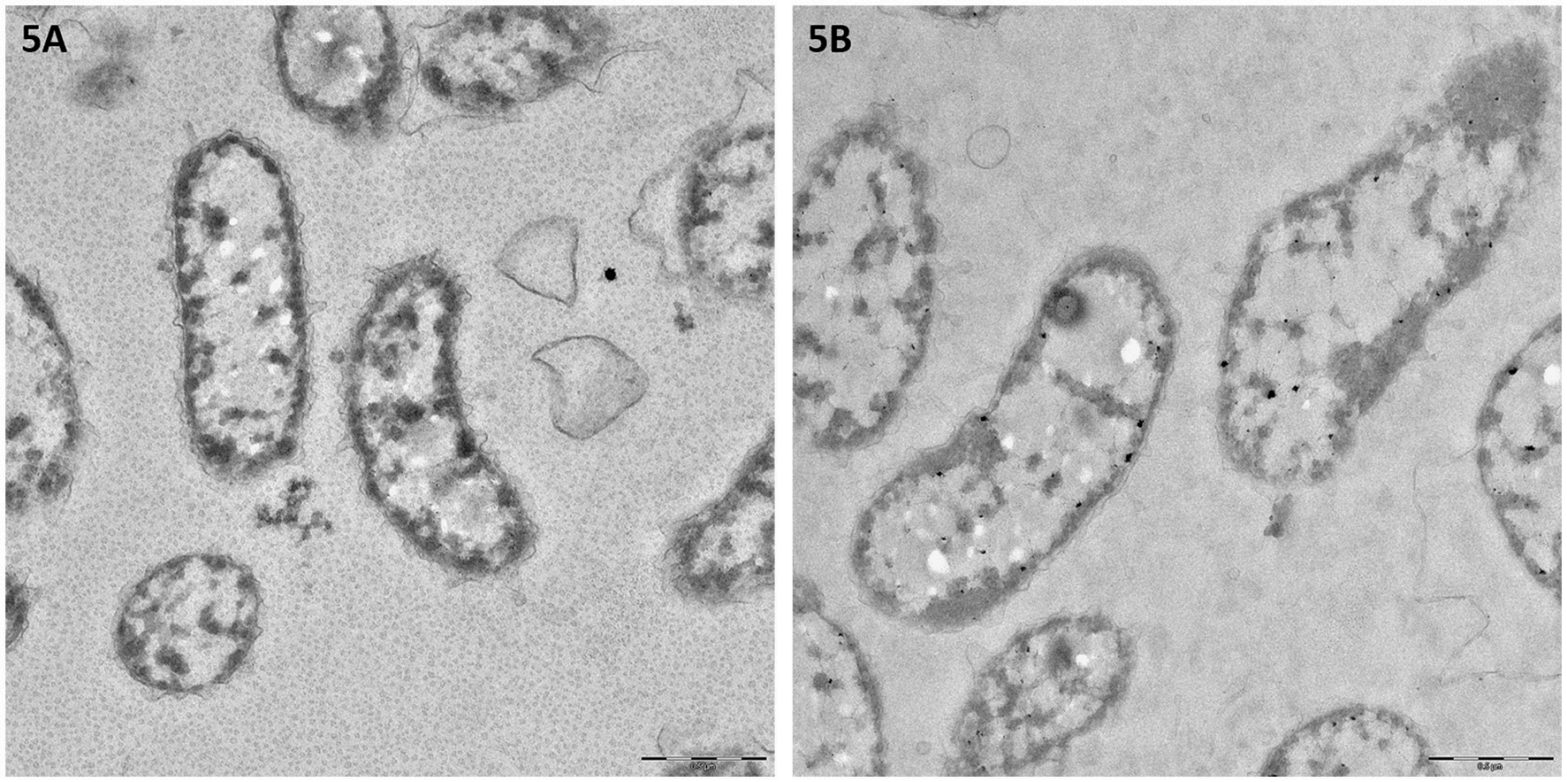
TEM images of *Vibrio anguillarum* after toxicity testing exposure in 2% NaCl saline solution: control **(A)** and CuO NP dispersion (40 mg/l, **B**).

The Transmission Electron Microscope (TEM) analysis (Figure 5) again did not highlight morphological differences between control and CuO NPs exposed bacteria. No evidence of internalization or adsorption were found. Some opaque very small particles are present on the biological material.

## Discussion

The bioassay with *V. anguillarum* allowed to assess the CuO NP toxicity and highlighted a significant variation of the toxic effect with salinity, depending on a different aggregation state of NPs.

According to the OECD guidelines (OECD, 2014), the CuO NP behavior in the control and exposure media has been accurately investigated, to link characterization data with ecotoxicological results; this allowed a correct interpretation of the response. In our study, through the analyses by DLS and LUMISizer, the aggregation and stability of the CuO NPs were verified at three different salinities of the exposure media. The DLS analysis (see Table 1 and Figure S1) highlighted a bimodal particle size distribution, with nanoscale (about 80 nm) and microscale (about 200 nm) aggregates, in both mQW and 0.5% saline solution; while, the dispersions in 2.0 and 3.5% saline solutions showed a single size population of microscale aggregates (250–300 nm). The low ζ-potential absolute values also confirmed the aggregation state and instability of the CuO NPs. Although Zeta (ζ-) potential is commonly considered a key parameter to describe the NP behavior in complex environmental media, it is worth to note that the high ionic strength of saline exposure medium, may shield electric charge of NPs, lowering the measured ζ-potential.

The LUMISizer analysis (see Figure 2) confirmed the characterization by DLS and provided an even more accurate representation of the particle size distribution in the exposure media. In fact, LUMISizer better characterized the big size nanoparticle populations: the presence of nano- and micro-scale aggregates in mQW and 0.5% saline solution was confirmed, and also the microscale aggregates in 2.0 and 3.5% saline solutions up to a size of about 1,000 nm were detected. Furthermore, the LUMISizer analysis allowed to quantify the sedimentation rates of CuO NP aggregates in the exposure media (see Figure S2). Only a negligible sedimentation of the CuO NPs as aggregates occurred during the exposure time (6 h), even at the highest salinity. This result was obtained with some dedicated features of the bioassay (i.e., test tube size, exposure volume and continuous agitation), designed to limit the NP sedimentation. Being inversely correlated with NP toxicity (Buffet et al., 2011; Villareal et al., 2014), the NP sedimentation rate represents a relevant parameter to describe the NP behavior in environmental media, particularly for salt water matrix. However, only few studies address the NP sedimentation rate and, at our best knowledge, this study is the only one that measured the sedimentation rates in seawater.

The particle diameter and sedimentation rate increased at increasing salt concentrations, due to the effect of ionic strength. Low salinity (mQW and 0.5% NaCl) allowed optimal and stable dispersion of CuO NPs, without differences between the two media. While, at high salinity (2.0–3.5% NaCl) agglomeration was promoted by the presence of salt ions, which shield the NP charge reducing the repulsive effect among NPs.

The increased particle size at high salinity determines a decrease of the total surface area; this implies a decrease of the superficial reactivity of NPs (because of agglomeration) which, in turn, produces a reduction of the toxic effects. The results obtained with this bioassay confirm that agglomeration and stability of CuO NPs are inversely related to their toxicity. At the lowest salinity (0.5% NaCl), when CuO NP agglomeration was the least and stability of dispersions was optimal, high toxicity (EC_50_ = 12.6 mg/l) was recorded, suggesting a particularly relevant hazard for these NPs when released in brackish habitats. At the highest salinity, typical of marine environments, the occurring aggregation and sedimentation suggest that Cu NPs may accumulate in sediments (Buffet et al., 2013) and therefore, benthic organisms are supposed to be the most exposed to NPs. However, the fate and bioavailability of NPs in sediments can be highly variable and depend on several abiotic and biotic factors, including ionic strength of water, amount of suspended natural organic matter (Keller et al., 2010), light intensity or temperature (Zhou et al., 2012) and biogenic transformation processes (decomposition, bioturbation, or digestion; Farré et al., 2009).

Table 3 summarizes the studies on CuO NP toxic effects on bacterial species; they show highly variable values of the Effect Concentrations, even in test on the same bacterial species/strain. This depends on the type of nanoparticles and on the experimental conditions (Bondarenko et al., 2013). This variability confirms the difficulty to compare the ecotoxicological assessments of nanomaterials, even among standardized bioassays, and highlights the need of highly reliable and reproducible tests.

**Table 3:** Comparison of EC_50_ and/or main results obtained for short- and long-term exposures to copper oxide nanoparticles (CuO NPs) on different bacterial species.

**Species**	**Exposure medium**	**Exposure time**	**Endpoint (inhibition of)**	**CuO NP size (nm)^a^**	**CuO NP EC_50_ (mg/l)**	**EC_50_ controls CuSO_4_, (CuObulk) (mg/l)**	**References**
*Bacillus subtilis*	LB agar	24 h	Growth	20–30^a^	61.1	–	Baek and An, 2011
*Bacillus subtilis*	LB	4 h	Growth	24.5 ± 2.3^c^ 152 ± 2^b^	>100	>100	Bondarenko et al., 2016
*Escherichia coli*	LB agar	24 h	Growth	20–30^a^	28.6	–	Baek and An, 2011
*Escherichia coli*	LB	4 h	Growth	24.5 ± 2.3^c^ 152 ± 2^b^	> 100	>100	Bondarenko et al., 2016
*Escherichia coli*	MMD	24h	Growth (OD)^5^	20–100^c^ 300 ± 2^b^	160	140 (>250)	Kaweeteerawat et al., 2015
*Escherichia coli* ^**^	HMM	8h	Bioluminescence (ROS induction/SS DNA breaks)	30^a^ 385^b^	6^*^	0.6 (600)	Bondarenko et al., 2012
*Lactobacillus brevis*	MRS	24 h	Growth (OD)^5^	20–100^c^ 470 ± 4^b^	3.6	24 (>250)	Kaweeteerawat et al., 2015
*Pseudomonas aeruginosa*	LB	4 h	Growth	24.5 ± 2.3^c^ 152 ± 2^b^	>100	>100	Bondarenko et al., 2016
*Pseudomonasputida*	LB	4 h	Growth	24.5 ± 2.3^c^ 152 ± 2^b^	>100	>100	Bondarenko et al., 2016
*Staphylococcus aureus*	LB agar	24 h	Growth	20–30^a^	65.9	-	Baek and An, 2011
*Staphylococcus aureus*	LB	4 h	Growth	24.5 ± 2.3^c^ 152 ± 2 ^b^	> 100	> 100	Bondarenko et al., 2016
*Vibrio fisheri*	2% NaCl	30 min	Bioluminescence (Flash test)	24.5 ± 2.3^c^ 152 ± 2^b^	4.3	0.3	Bondarenko et al., 2016
*Vibrio fisheri*	2% NaCl	30 min	Bioluminescence	30^a^	79	1.6 (3,811)	Heinlaan et al., 2008
*Vibrio fisheri*	2% NaCl	30 min	Bioluminescence (Flash test)	30^a^	68.1 (cuve) 204 (plate)	2.0 (3,894)	Mortimer et al., 2008
*Vibrio fisheri*	2% NaCl	30 min	Bioluminescence	30–40^a^ 302 ± 31.37^b^	257 mg/L	(1,472)	Rossetto et al., 2014

a *Declared*.

b *Measured in test solution (hydrodynamic diameter)*.

c *Primary size (TEM)*.

* mg Cu/l

** *Recombinant strains, bioassays performed in 96 well-microplate*.

The bioassay with *V. anguillarum* clearly showed a significant decrease of bacterial culturability, at increasing concentration of CuSO_4_ 5H_2_O and CuO bulk, used in this study as solubility and size controls, respectively. Unlike CuO NPs, size and solubility controls did not elicit different toxicities at different salinities. As expected, a clearly different toxicity was observed among the three copper forms; according with the EC_50_ values, toxicity ranking is as follows: CuSO_4_ 5H_2_O > CuO NP > CuO bulk. CuO NPs toxicity is an order of magnitude lower than CuSO_4_ 5H_2_O but higher than CuO bulk. Importantly, the EC_50_ values for CuSO_4_ 5H_2_O are comparable with those obtained by acute tests on other marine species (Adams and Stauber, 2004; Manfra et al., 2015; Rotini et al., 2018) including bacteria (see Table 3); this demonstrates the good sensitivity of our recently proposed bioassay for both conventional contaminants and NPs.

It is generally accepted that CuO NP toxicity is higher than CuO-bulk, depending on size, surface characteristics, dissolution, and exposure routes (reviewed by Chang et al., 2012), on the contrary, the contribution of dissolved Cu^2+^ ions to the observed toxicity of CuO NPs is still under discussion (Ivask et al., 2013; Gonçalves et al., 2017). Several studies ascribe primarily to the Cu^2+^ ion dissolution the CuO NP toxicity in biological systems (Heinlaan et al., 2008; Aruoja et al., 2009; Kahru and Dubourguier, 2010; Mortimer et al., 2010; Bondarenko et al., 2012; Kasemets et al., 2013). A solubility-dependent toxicity of CuO NPs has been observed in *Daphnia magna* (Heinlaan et al., 2008; Blinova et al., 2010; Fan et al., 2012; Jo et al., 2012), *Cyprinus carpio* (Zhao et al., 2011) and zebrafish embryos (Lin et al., 2015). On the contrary, just as many other studies found that the toxic effects of CuO NPs do not depend from the ion dissolution (Heinlaan et al., 2008; Jiang et al., 2009; Baek and An, 2011; Isani et al., 2013). Agreeing with this last group, our results indicate a modest Cu^2+^ dissolution with an inverse salinity-dependence; as a consequence, the toxic effects observed for CuO NPs might be due to intrinsic toxicity mechanisms related to the nano-form as, for instance, aggregation plays a key role. This was already suggested by other authors (Buffet et al., 2013; Gonçalves et al., 2017), although the issue deserves further investigations. Both SEM and TEM analyses did not evidenced morphological differences between control and CuO NPs exposed bacteria. Interestingly, the SEM images (see Figure 1) showed two surface structures present on both control and exposed bacteria: outer membrane vesicles (*a.k.a*. blebs), known to be unique for *Vibrio* strains, and fibrils. Blebs and fibrils are signature of starvation and are produced by the bacterial cells in response to the growth arrest (Östling et al., 1993). This response was expected because during the test bacteria are exposed to the toxicant in the absence of nutrients. The TEM images again do not suggest CuO NP internalization into or intimate adhesion to bacteria. Some opaque particles seem to be included into the bacterial cells, but their small size and shape does not support their identification as the NPs used in this study. Hence, our results do not chime with the evidence by Kaweeteerawat et al. (2015), which found nano Cu species strongly bound to or internalized within *E. coli* cells and stated that both nano Cu and nano CuO can be internalized into the bacterial cell. Probably the different findings can be ascribed to the size of NPs and their aggregates that, in our study, are not compatible with internalization into a *Vibrio* bacterial cell.

The bioassay with *V. anguillarum* benefits from several procedural points in assessing the NP toxicity. As a first point, it assesses culturability, *i.e*. the bacterial capability to actively replicate and form colonies after the exposure to NPs in a saline medium. The endpoint culturability allows an easier comparison of results with those from the most common ecotoxicological bioassays for marine environments, which often have survival/mortality as endpoint; this will facilitate its introduction in test batteries. Furthermore, it is interesting to highlight that the reduction of bacterial culturability can be also due to the reversible VBNC (Viable But NonCulturable) state which is known to be induced/triggered by a variety of stressors such as out range of growth temperature, oxygen concentrations, heavy metals, etc. (Oliver, 2005, 2009), hence increasing the sensitivity of the test. Moreover, the exposure is carried out in simple saline solution, avoiding any possible interferences of nutrients with the NPs, known to modify the reproducibility of results by increasing or reducing NP bioavailability (Ivask et al., 2013; Kasemets et al., 2013). Again, the short time of exposure (6 h) is long enough to observe and evaluate acute effects on the bacterial population and short enough to limit sedimentation and aggregation of metal NPs in salt water, ensuring repeatability of the exposure conditions. Last but not least, this bioassay has no limitations for colored/turbid samples, and is applicable on a wider range of salinities, common limitations of the existing methods with microorganisms. This feature of the bioassay gives the possibility to carry out contemporary assays in a wide range of salinity (0.5–3.5%), which is known to affect NP behavior and toxicity (Corsi et al., 2014).

## Conclusion

In this study, a recently designed bioassay with the marine bacterium *V. anguillarum* allowed to assess the toxic effects of CuO NPs, showing a clear dose-response relationships and a crucial role of salinity and particle aggregation in the observed toxicity. Results highlighted the high toxicity of CuO NPs, particularly at low salinity, and pointed out a relevant hazard for these NPs in brackish environments. While, at salinities typical of marine environments the high aggregation and sedimentation rate of these NPs suggest a possible accumulation in sediments, increasing the risk of exposure for benthic organisms. The influence of salinity on the NP toxicity is a hitherto little explored issue and further ecotoxicity assessments, including different NPs and bioassays are particularly needed. Moreover, our results demonstrated the effectiveness of *V. anguillarum* bioassay as a promising tool for the risk assessment of nanomaterials and confirmed its useful application on conventional contaminants too.

## Author contributions

LM, AR, MT, and LoM conceived and designed the study. AR, AT, and LoM performed ecotoxicological analyses. MB, CM, and RC performed chemical analyses. FI and GB performed microscopy. MT choose the suitable *Vibrio* strain and the cultural conditions. AR, AT, and LoM wrote the manuscript; all authors contributed to the discussion and approved the final manuscript.

### Conflict of interest statement

The authors declare that the research was conducted in the absence of any commercial or financial relationships that could be construed as a potential conflict of interest.
