# Supplementary material for: Salinity-Based Toxicity of CuO Nanoparticles, CuO-Bulk and Cu Ion to Vibrio anguillarum
Source: Front Microbiol. 2017 Oct 25;8:2076. doi: 10.3389/fmicb.2017.02076 (PMC5661029; doi:10.3389/fmicb.2017.02076)
Supplement: Supplementary file 1 [file Presentation1.PDF]

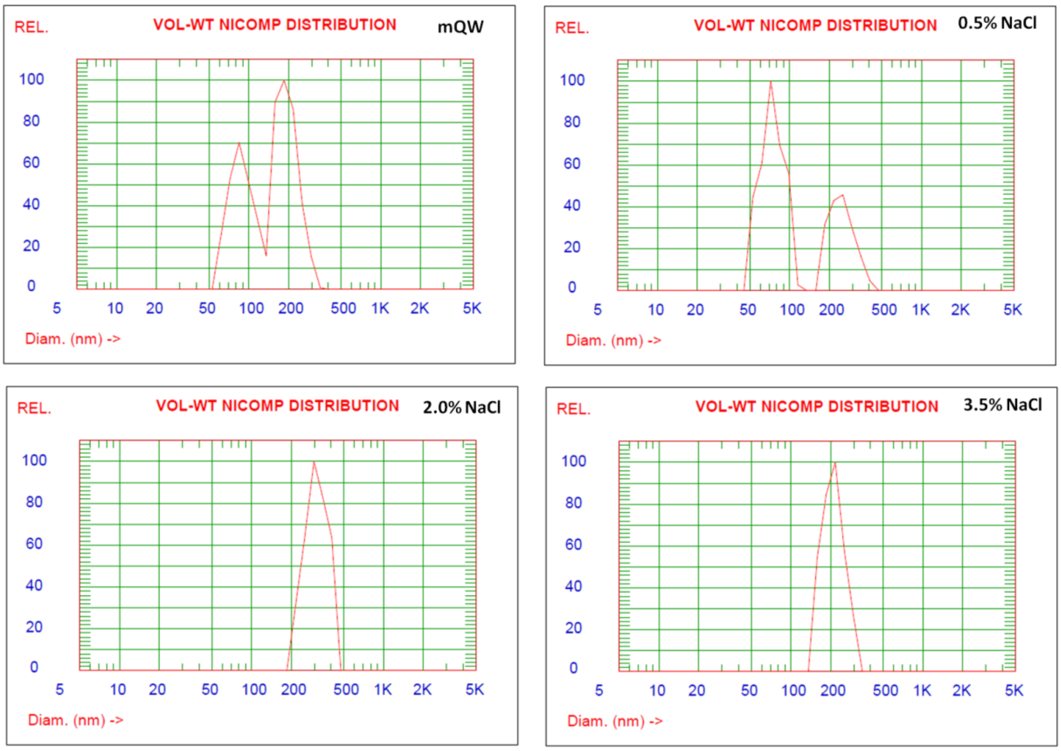

Figure S1| Volume-weighted (VOL-WT) size distribution (nm) of CuO NP dispersions in milli-Q water used as reference medium (mQW, T = 25°C, 0% NaCl) and three saline solutions used as exposure media (T = 25°C, 0.5-2.0-3.5% NaCl) using DLS analysis, 3h after sonication. Distributions refer to 40 mg/l of CuO NP.

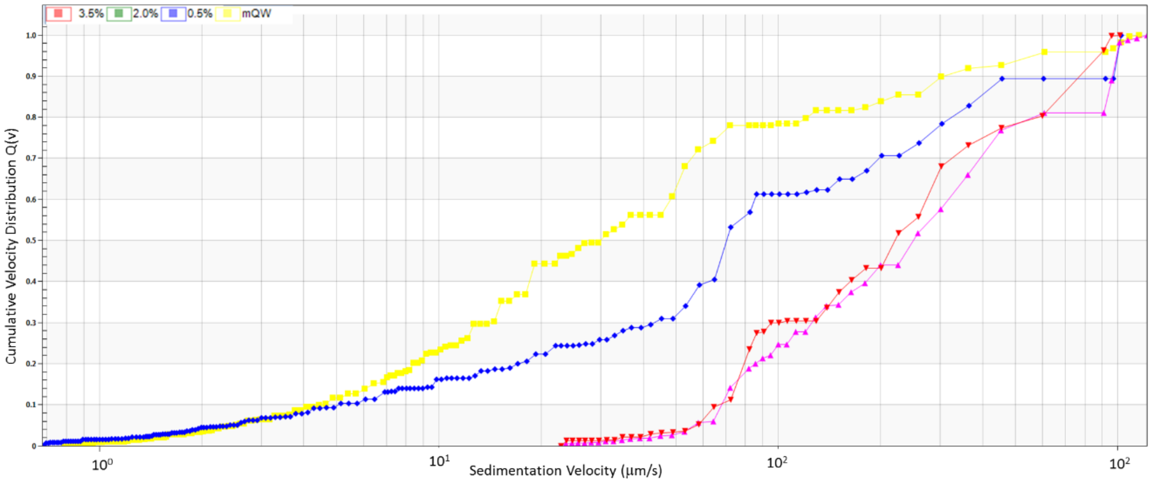

Figure S2| Sedimentation velocity profiles of CuO NPs in milli-Q water used as reference medium (mQW, T = 25°C) and in three saline solutions used as exposure media (T = 25°C, 0.5-2.0-3.5% NaCl).

Table S1| Cu^{2+} dissolution at 40 mg/l of CuO NP suspension at increasing NaCl concentrations (T = 25°C, 0.5-2.0-3.5% NaCl). CuO NP final suspension was centrifuged (4000*g*; 60 min) to remove the non-soluble fraction. Values are the mean value (± SD) of 3 measurements.

Sample	Dissolved Cu^{2+} (µg/l, ± SD)	Cu^{2+}dissolved/CuO NP (%)
Control	0.618 ± 0.003	
0.5% NaCl+CuO NP	377.51 ± 4.26	1.2
2.0% NaCl+CuO NP	153.47 ± 3.54	0.5
3.5% NaCl+CuO NP	215.01 ± 1.27	0.7

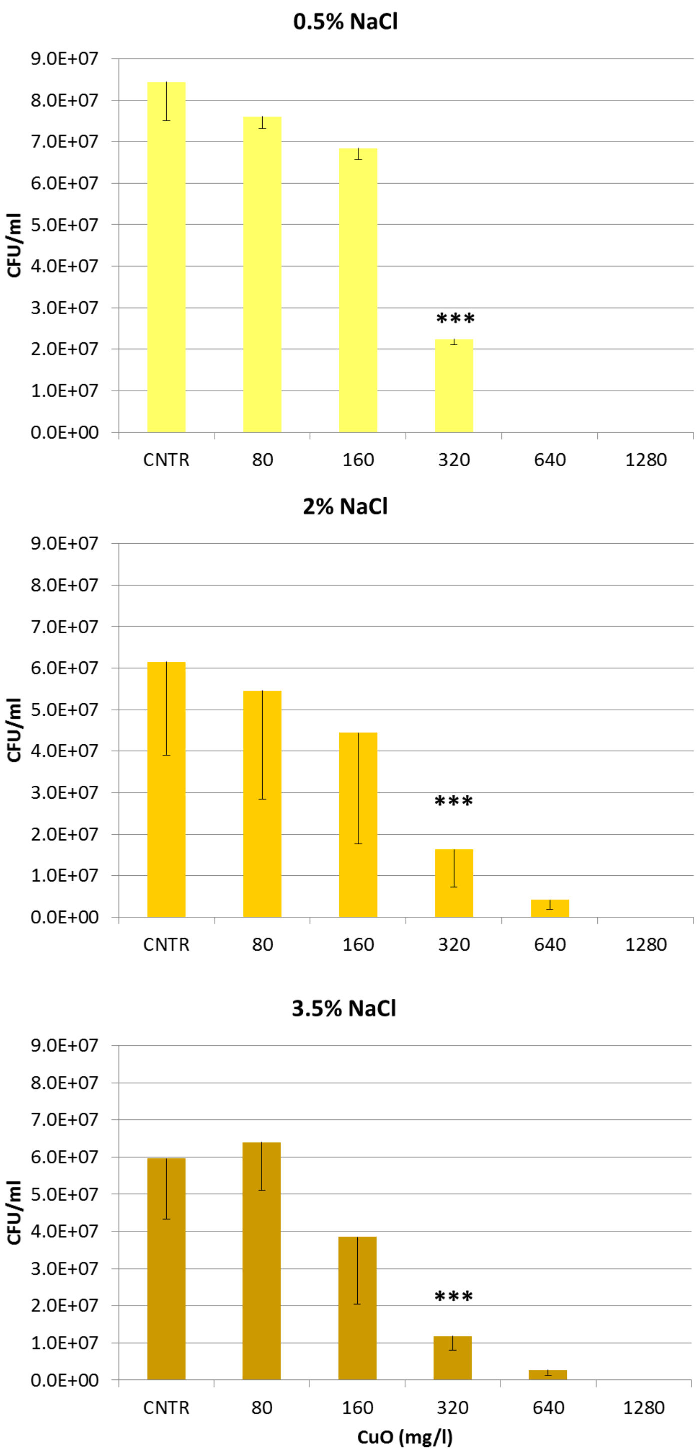

Figure S3| Mean number of CFU/ml (CFU=Colony Forming Unit) of *Vibrio anguillarum* after 6-hr exposure to different concentrations of CuO (mg/l) at three different salinity of the exposure medium (0.5-2.0-3.5% NaCl). Values represent the mean (±SD) of three independent trials. Significant reduction of CFU/ml compared to control, based on *post-hoc t*-test, are indicated with asterisks (**=p<0.01;***=p<0.001).

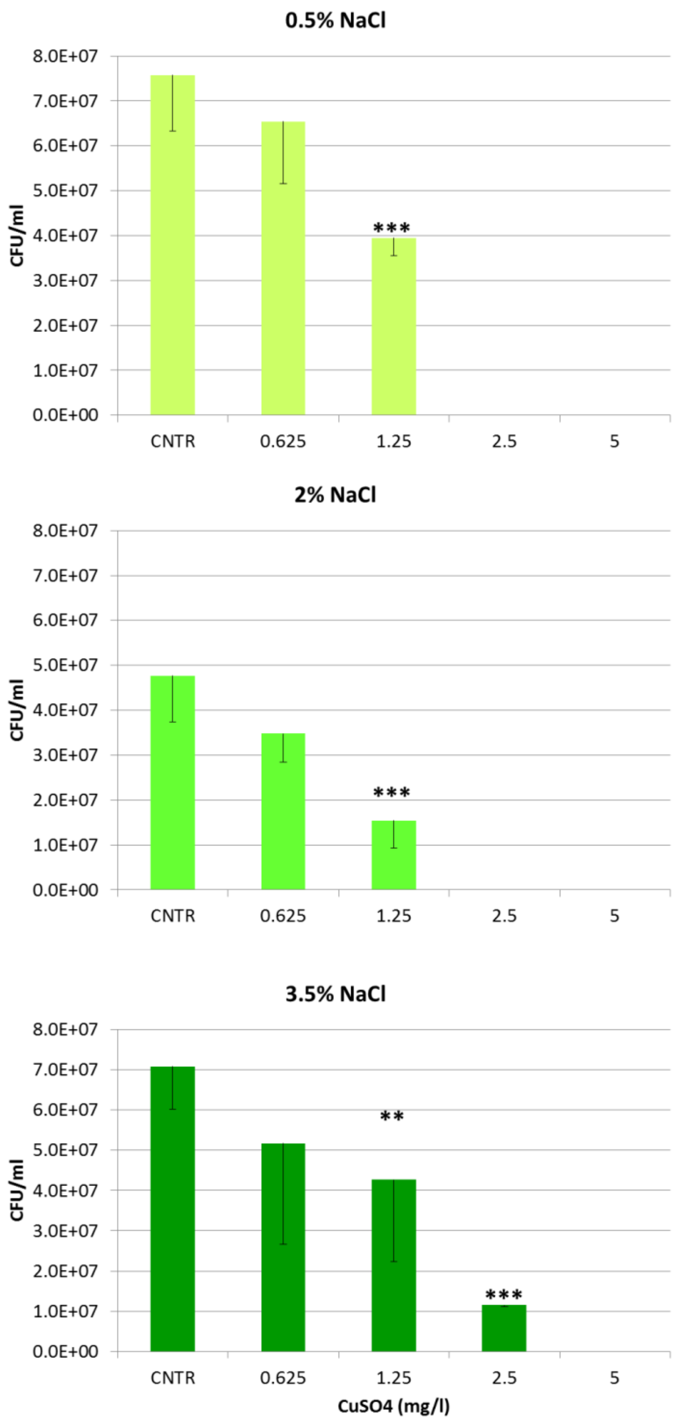

Figure S4| Mean number of CFU/ml (CFU=Colony Forming Unit) of *Vibrio anguillarum* after 6-hr exposure to different concentrations of $CuSO_4$ $5H_2O$ at three different salinity of the exposure medium (0.5- 2.0-3.5% NaCl). Values represent the mean (±SD) of three independent trials. Significant reduction of CFU/ml compared to control, based on *post-hoc t*-test, are indicated with asterisks (**=p<0.01;***=p<0.001).
